# Isolated Left Bundle Branch Block in a Toddler

**DOI:** 10.1155/2014/464579

**Published:** 2014-05-18

**Authors:** Hitesh Agrawal, Frank Zimmerman, Zahra Naheed

**Affiliations:** ^1^Division of Pediatrics, Department of Pediatrics, John H. Stroger Jr. Hospital of Cook County, 1900 W. Polk Street, Room 1134, Chicago, IL 60612, USA; ^2^Electrophysiology, Advocate Children's Hospital, 440 W. 95th Street, Oak Lawn, IL 60453, USA; ^3^Division of Pediatric Cardiology, John H. Stroger Jr. Hospital of Cook County, 1900 W. Polk Street, Room 1123, Chicago, IL 60612, USA

## Abstract

Left bundle branch block (LBBB) usually occurs as a postoperative complication from surgical correction of congenital heart disease and can be associated with hypertensive heart disease, coronary artery disease, myocarditis, and aortic valvular disease. Although isolated LBBB is a conduction abnormality found in some healthy adults, it has not been reported in pediatric population. We report a 2-year-old, healthy African American female who was incidentally discovered to have isolated LBBB that has persisted in a follow-up of 3 years.

## 1. Introduction

Isolated LBBB has been widely described in adult literature [1–5]. Previous studies have postulated that it is an independent predictor of mortality and confers a risk similar to that of conventional cardiac risk factors [4, 5]. However, electrocardiographic (EKG) analysis of large cohorts of healthy children has failed to identify this entity [6–8]. This discrepancy may be due to the fact that adults are predisposed to age related degeneration of the conduction system or may have undetected ischemic or valvular heart disease or cardiomyopathy [1], which is uncommon in children. Chiu et al. studied cardiac conduction disturbances in 432,166 children (age group 6–20 years) and mentioned 1 case with isolated LBBB. However no further description or follow-up of this case is mentioned in the article [8].

## 2. Case Report

**Figure 1:**
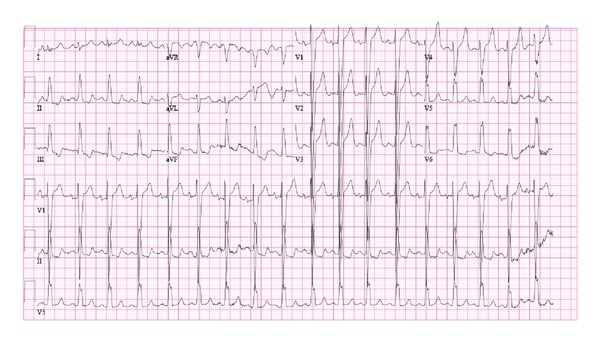
EKG tracing at presentation showing left bundle branch block with heart rate of 108/minute, PR interval of 148 milliseconds, QRS duration of 124 milliseconds, and QTc of 413 milliseconds.

**Figure 2:**
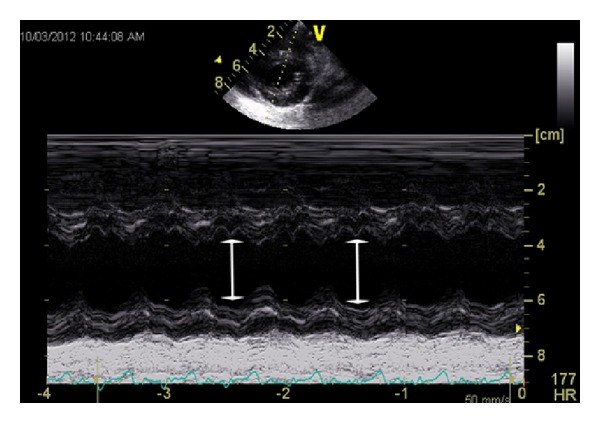
M-mode echocardiographic image, at 1-year follow-up, showing asynchronous motion of the interventricular septum during diastole, marked by arrows.

A 27-month-old African American female was brought to the emergency room within an hour of a questionable exposure to 1-2 pills of sustained release Nifedipine 30 mg tablets. Two tablets of Nifedipine were found missing in the grandmother's medication bottle following which she performed blind finger sweeps and retrieved some partially dissolved pill fragments from the patient's mouth. The child was alert and in no distress. Vitals' signs were stable: temperature: 98.1°F, heart rate: 109/minute, respiratory rate: 26/minute, and blood pressure: 116/69 mmHg. Cardiac exam revealed normal S1 and S2 with no murmurs. Initial laboratory studies including complete blood count, basic metabolic profile, and urinalysis were normal. No toxins were detected on urine toxicology screen. Activated charcoal of 1 gm/kg without sorbitol was given orally. Electrocardiography (EKG) demonstrated LBBB (Figure 1) with heart rate of 108/minute, PR interval of 148 milliseconds, QRS duration of 124 milliseconds, and QTc of 413 milliseconds. An echocardiogram demonstrated normal structural anatomy but M-mode showed asynchronous motion of the interventricular septum (Figure 2).

**Figure 3:**
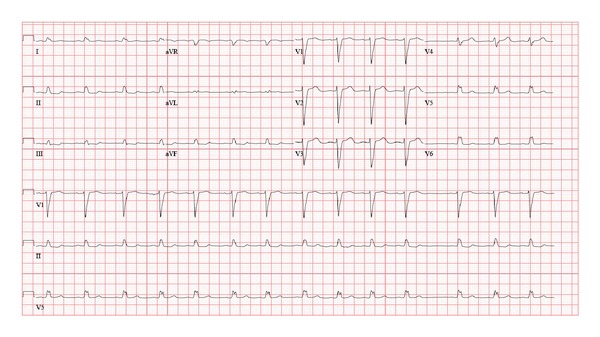
EKG tracing at 3-year follow-up showing persistence of left bundle branch block with heart rate of 84/minute, PR interval of 142 milliseconds, QRS duration of 126 milliseconds, and QTc of 377 milliseconds.

The child was admitted to pediatric intensive care unit for overnight observation. She remained hemodynamically stable and was discharged from the hospital the next day. Electrophysiology study done a year later showed a mildly prolonged H-V interval of 52 milliseconds (normal <50 milliseconds) and normal A-H interval of 54 milliseconds with otherwise normal AV conduction. No accessory pathways or dual AV nodal physiology was identified. No arrhythmias occurred with the induction protocols during the study. A 3-year follow-up with repeated EKGs (Figure 3) and echocardiograms showed that the LBBB persisted with preserved cardiac function. The ejection fraction has remained unchanged at approximately 60% (calculated via Simpson's method and M-mode), and E-point septal separation (EPSS) is <4.6 mm. The patient has remained asymptomatic since the initial diagnosis.

## 3. Discussion

Although Nifedipine poisoning causing bundle branch block and third degree heart block has been reported in children [9, 10], these are transient and occur at much higher doses—approximately 70 mg/kg [9]. Since the maximum possible dose of ingested Nifedipine was minimal in our patient and is much lower than the recommended therapeutic dose range of 0.5–3 mg/kg/day for management of pediatric hypertension [11], it was an unlikely etiology for the isolated LBBB.

Further testing with M-mode echocardiography showed asynchronous motion of the interventricular septum concurrent with the literature in adults with isolated LBBB. Grines et al. [12] postulated that LBBB causes a delay in the left ventricular depolarization resulting in delayed left ventricular contraction and relaxation compared with the right ventricle. This in turn can lead to diminished contribution of septal contraction towards ejection fraction and eventually result in systolic and diastolic dysfunction. However, this may not be prominent in childhood and may manifest later with age related degeneration of the conduction system.

## 4. Conclusion

A follow-up of 3 years has shown that patient has been asymptomatic and no progressive changes in the EKGs and echocardiograms have occurred. Hence, LBBB may be found as an isolated lesion in apparently healthy children even as young as 2 years of age. However, long term follow-up of cardiac conduction and ventricular function with serial EKGs and echocardiograms is required.
